# Cytochromes P450 of *Caenorhabditis elegans*: Implication in Biological Functions and Metabolism of Xenobiotics

**DOI:** 10.3390/biom12030342

**Published:** 2022-02-22

**Authors:** Lucie Larigot, Daniel Mansuy, Ilona Borowski, Xavier Coumoul, Julien Dairou

**Affiliations:** 1 Campus Saint Germain, INSERM UMR-S 1124, Université de Paris, 45 rue des Saints-Pères, 75006 Paris, France; luciole271@gmail.com; 2 Laboratoire de Chimie et de Biochimie Pharmacologiques et Toxicologiques, CNRS, Université de Paris, 75006 Paris, France; daniel.mansuy@parisdescartes.fr (D.M.); ilona.borowski@etu.u-paris.fr (I.B.)

**Keywords:** cytochrome P450, worm nematode, predictive toxicology, xenobiotics

## Abstract

*Caenorhabditis elegans* is an important model used for many aspects of biological research. Its genome contains 76 genes coding for cytochromes P450 (P450s), and few data about the biochemical properties of those P450s have been published so far. However, an increasing number of articles have appeared on their involvement in the metabolism of xenobiotics and endobiotics such as fatty acid derivatives and steroids. Moreover, the implication of some P450s in various biological functions of *C. elegans*, such as survival, dauer formation, life span, fat content, or lipid metabolism, without mention of the precise reaction catalyzed by those P450s, has been reported in several articles. This review presents the state of our knowledge about *C. elegans* P450s.

## 1. Introduction

The soil nematode *Caenorhabditis elegans* (*C. elegans*) is one of the simplest organisms with a laboratory model status. *C. elegans* is a transparent worm of about 1 mm in length, found in temperate soil environments [1]. *C. elegans* is an important model used for many aspects of biological research [2]. It is a non-infectious and non-pathogenic organism that survives by feeding on microbes such as bacteria. With abundant food and low population density, *C. elegans* has a lifespan of around two or three weeks with a generation time average of three and half days. *C. elegans*’s developmental stages are eggs, larvae, fertile adults, and post-reproductive adults. *C. elegans* larvae complete development from embryo to adult with four larval stages (L1–L4) in three days. However, in an environment with limited food and/or high population density, larvae may arrest development at L2 to enter a particular stage called the dauer stage (L2d [3]). The dauer larva has a unique morphology with physiology and metabolism, which allow resistance to environmental stress. The dauer larva can live up to several months, and this stage ends when conditions favor further growth of the larva, now into the L4 stage. The dauer stage is considered to be non-aging because the post-dauer life span is not affected by the length of this stage [4]. The *C. elegans* model proposes the power of integrated whole animal investigations that are cost- and time-efficient and require minimal infrastructure. It is nowadays a powerful model organism not only for developmental biology but also for aging studies or toxicology [2,5]. *C. elegans* somatic cell locations and types, as well as networks neurons, have been mapped [6], allowing morphological evaluations of abnormalities induced by toxins and deep neurological/behavioral correlations. Furthermore, genes and signaling pathways appear to be well conserved between *C. elegans* and humans [7,8]. Despite their different complexity, the number of genes in *C. elegans* and humans is surprisingly similar (~21.000 genes in humans, ~19.000 genes in *C. elegans*) [9].

Contrary to toxicity tests using cell cultures, *C. elegans* toxicity tests provide data from a whole animal with intact and metabolically active digestive, reproductive, endocrine, sensory, and neuromuscular functions. Toxicity ranking, including LD50, in this nematode, has repeatedly been shown to be as predictive as the LD50 rankings using rats or mice [5]. However, the defense mechanisms against xenobiotics in *C. elegans* have been little studied, although they are essential in toxicological studies. The xenobiotic-metabolizing enzymes, and particularly P450-dependent monooxygenases (phase I enzymes), play a key role in these defense mechanisms. In this review, we present the state of our knowledge about *C. elegans* P450s.

P450s are ubiquitous heme-thiolate proteins involving iron-protoporphyrin IX in their active site that are widely distributed in living organisms [10,11]. The common property to all P450s discovered so far is the peculiar position of the Soret peak of their Fe(II)–CO complex around 450 nm. This redshifted Soret peak is the signature of the presence of a cysteinate ligand from the protein bound to the iron in trans position to the CO ligand [11]. Most P450s catalyze monooxygenase reactions, such as hydroxylations or epoxidations, on a great number of substrates. These reactions require the presence of dioxygen as well as a cofactor, NADPH or NADH, providing the necessary electrons via electron transfer proteins that are very often coupled to P450 inside cell membranes [11]. In the human genome, 57 genes encoding P450s have been identified (drnelson.uthsc.edu (accessed on 24 January 2022)). Some P450s catalyze oxidation steps involved in the biosynthesis and/or biodegradation of endogenous compounds such as steroids, fatty acids, and endocannabinoids [12]. On the other hand, P450s play a key role in the oxidative biotransformation of xenobiotics such as drugs, pesticides, and other environmental chemicals, facilitating their elimination from living organisms [11,12].

We know of over 62,000 bacterial P450s and 85,000 fungal P450s [13]. Since 1989, P450s have been classified according to their degree of identity in their amino acid sequence. Indeed, P450s with a degree of identity greater than 40% belong to the same family and are designated by a number behind the abbreviation P450 or CYP (CYP13, CYP14). When they have a degree of identity greater than 55%, they belong to the same subfamily and are therefore designated by a capital letter behind the number of the family (CYP13A, CYP13B). Finally, isoforms belonging to the same subfamily are differentiated by a number behind the letter of the subfamily (CYP13A1, CYP13A10). Humans have 57 functional P450s classified in 18 families and 43 subfamilies [12,14]. The largest P450s families in humans are 2, 3, and 4, and some of them are even larger in other mammals such as a mouse. As the number of families increased, it was necessary to establish a new reunification. Clans bring together families that belong to the same group (from the same ancestral gene) according to many phylogenetic trees established previously [15]. There are 11 animal P450s clans (clans 2, 3, 4, 7, 19, 20, 26, 46, 51, 74, and mitochondrial or mito). However, not all organisms own all 11 clans. For example, ecdysozoa (insects, crustaceans, nematodes including *C. elegans*) only have clans 2, 3, 4, and mito [15,16], whereas humans have 10 out of 11 clans, all except clan 74 [17].

The genomic sequence of the nematode *C. elegans* reveals over 19,000 genes, of which 76 encode for P450s [9]. Studies on this subject, having led to about a hundred articles that will be mentioned below, show that some P450s are involved in the regulation of the transition to the dauer state as well as other physiological functions of the worm. Other P450s are involved in the metabolism and bioactivation or detoxication of xenobiotics. As vertebrates, *C. elegans* can induce some P450s, in particular through the Aryl hydrocarbon receptor (AhR) signaling pathway. AhR is a receptor well known for its fundamental role in the metabolism of xenobiotics in vertebrates [18]. Ligands of AhR are hydrophobic xenobiotics including polycyclic aromatic hydrocarbons and halogenated compounds such as benzo (a) pyrene and 2,3,7,8-tetrachlorodibenzo-*P*-dioxin. AhR also has endogenous ligands such as some steroids and kynurenine. Activation of AhR leads to its translocation from the cytoplasm to the nucleus and then its heterodimerization with its partner, the aryl hydrocarbon receptor nuclear translocator (ARNT), thus forming a transcription factor. The heterodimer AhR/ARNT directly regulates the expression of many genes, including those of some enzymes involved in the metabolism of xenobiotics (P450s of family 1 and glutathione-*S*-transferases) [19].

In the following, we will review what is presently known about *C. elegans* P450s.

## 2. Genetic and Phylogenetic Analysis of *C. elegans* P450s

*C. elegans* was the first multicellular organism to have its whole genome sequenced [9] and is the only organism to have its connectome (neuronal “wiring diagram”) completed [20]. It contains 82 P450 genes, including 6 pseudogenes, divided into 16 families (13, 14, 22, 23, 25, 29, 31, 32, 33, 34, 35, 36, 37, 42, 43, 44) and 26 subfamilies in accordance with the Nelson’s nomenclature [21]. Table 1 shows all the *C. elegans* P450s grouped by family. Almost all of the *C. elegans* P450s families appear to be nematode-specific [22], but they correspond to the clans 2, 3, 4, and mito also found in humans [16].

Some families have only one member (22, 23, 36, 42, 43, and 44) while others are very large, such as family 33 with 18 P450s (Table 1).

To compare the *C. elegans* and human P450s, a phylogenetic analysis was made. In order to achieve a phylogenetic tree, the sequences of all P450s (humans and *C. elegans*) were collected from the Uniprot website [23]. All trees were produced thanks to the Phylogeny site [24]: the P450 tree of *C. elegans*, that of human P450s, then a tree grouping together all these P450s. Subsequently, the creation of a more visual tree was carried out with the Cytoscape software [25].

**Figure 1:**
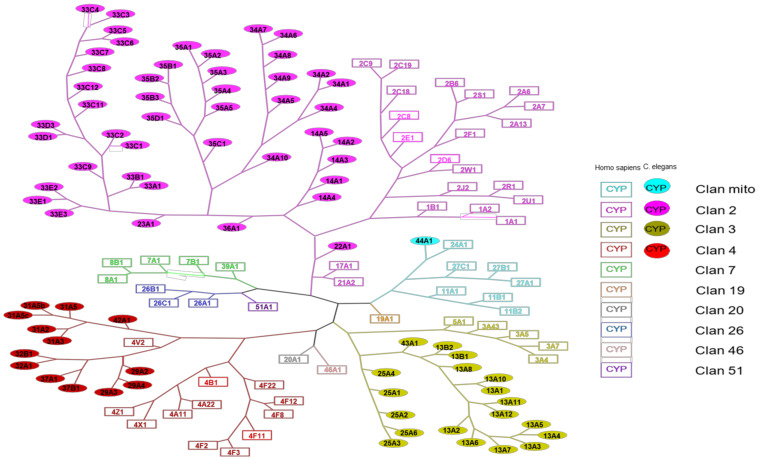
Phylogenetic tree gathering human and *C. elegans* P450s. The square boxes represent human P450s, while the filled round boxes represent *C. elegans* P450s. Different colors are assigned according to the clan to which the CYP450s belong.

The resulting phylogenetic tree common to humans and *C. elegans* P450s, shown in Figure 1, indicates interspecies relationships.

Human P450s belong to 10 different clans, whereas *C. elegans* P450s only belong to 4 clans. Some sequences of *C. elegans* P450s are closer to human P450s sequences than to those of other *C. elegans* P450s: this is particularly the case for P450s 22A1 (clan 2, close to 17A1 and 21A2) and 44A1 (clan mito, close to 24A1). This also occurs the other way around, as for human P450 4V2 (clan 4) that is found on the tree more closely related to *C. elegans* P450s 29A, 32, and 37 than to other human P450s of the same family (P450s 4B1, 4F22). P450 13A10 is found in clan 3. It belongs to the same clan as P450 3A4, which is one of the most important human P450 quite often involved in the metabolism of xenobiotics [12]. We also distinguish differences in the distribution of P450s in the clans between humans and *C. elegans*. Indeed, although clans 2 and 3 present a proportion rather like that expected considering the higher number of P450s in *C. elegans* than in humans, we can notice that only one of the 76 P450s of the nematode has a mitochondrial sequence. The proportion is, on the other hand, very different for humans who have in this clan 7 P450s out of 57 in total. Clan 4 also has a higher percentage of human P450s (12 P450s out of 57 in total) than *C. elegans* P450s (13 P450s out of 76 in total). In these two species, the largest P450s clan is clan 2, whereas, in insects, clan 3 is the largest one [22].

P450s can also be classified on an even higher level into groups that contain similar P450 clans across animals, plants, and bacteria [26]. P450 families have emerged and been lost during evolution. For example, P450 51A1 (Sterol 14α-demethylase) required for one step of cholesterol synthesis is evolutionarily old and found in bacteria, plants, and humans. However, it is not found in nematodes or insects that do not synthesize cholesterol de novo. Most of the phylogenetically well-preserved P450 families typically have only one or a few members conserved across many species, whereas unstable P450 families have variable numbers of members in different species. It is believed that the stable P450 families are essential in the synthesis or degradation of endogenous substrates, while the highly diverse P450 families metabolize xenobiotics or secondary metabolites [27,28].

## 3. Implication of P450s in Biological Functions of *C. elegans*

Most of the physiological roles of P450s in *C. elegans* have been determined by the invalidation of P450 expression (knock-out worms or knock-out/gene silencing by RNA interference). Thus, the implication of some P450s in the biological functions of *C. elegans* without mention of the precise enzymatic reactions catalyzed by those P450s has been reported in several articles (Table 2).

The silencing of single P450 genes has been described to cause alterations in survival [34,35,37], life span [30,31,32,33,34,35,36], morphology [36,37,38,39,40,41,62], embryonic development [37,42,43], larval development [35,37,42,44], dauer formation [29,30,31,35,45,46,47,48,49,50], reproduction [31,42,51,52,53], fat content [42,47,48,51,53,54,55,56], or lipid metabolism [32,40,42,47,48,50,51,54,55,57,58,59,60,61].

Molecular analyses from several studies allowed a better characterization of the roles of some P450s. It was proposed that P450-dependent eicosanoids may serve as second messengers in the regulation of pharyngeal pumping and food uptake in *C. elegans* [63]. Some *C. elegans* P450s are linked to the metabolism of fatty acid-derived signaling molecules [47,55]. The silencing of P450s 31A2 and 31A3 leads to polarization and osmotic defects and to failures in meiosis and embryonic development [42]. P450s 31A2 and 31A3 also appear to be involved in the biosynthesis of lipids that are essential for the correct formation of eggshells [42]. In addition, P450 31A2 is required for sperm motility [64], and it negatively regulates the synthesis of prostaglandins [51]. Similarly, the P450s of the 35A family regulate the levels of several fatty acids and endocannabinoids in *C. elegans* [54]. Thus, P450s 35A2 and 35A3 are involved in the production of lipids required for eggshell formation [42]. Moreover, the deletion of P450 35A2 affects the lipid regulation and longevity of *C. elegans* [32]. It was also shown that the hypoxia inducible factor, HIF, regulates *C. elegans* stress responses and behavior via the nuclear receptor NHR-46 by targeting the gene coding for P450 36A1 [65]. This suggests that P450 36A1 is involved in the biosynthesis of a hormone ligand for this receptor.

Interestingly, there are several P450s that have been implicated in an adaptive response to changing environmental conditions. For example, Cong et al. found six P450 genes (13A8, 13A11, 14A4, 33C2, 33D3, and 35B2), whose expression levels were very low at pH 6.33, while these genes were significantly upregulated when the pH dropped to 3.13 [66].

Only a small number of reactions catalyzed by *C. elegans* P450s and involving endogenous substrates have been identified so far (Table 3).

Thus, microsomes from adult worms oxidize eicosapentaenoic acid, EPA (a polyunsaturated fatty acid that acts as a precursor of prostaglandins, eicosanoids, and tromboxanes), with the main formation of 17,18-epoxy-eicosatetraenoic acid, 17,18-EEQ [47]. This oxidation is NADPH, and cytochrome P450 reductase dependent and is inhibited by usual inhibitors of mammalian arachidonic acid-metabolizing P450s. RNAi gene silencing experiments showed that P450s 29A3 and 33E2, which are related to mammalian P450 family 2, are mainly responsible for the oxidation of EPA into 17,18-EEQ [47]. Further studies using *C. elegans* P450 33E2 and human P450 reductase expressed in insect cells showed that P450 33E2 catalyzes the epoxygenation of EPA with the formation of 17,18-EEQ and the hydroxylation of arachidonic acid, AA, into 19-hydroxy-AA [55]. This article also showed that P450 33E2 is expressed in the *C. elegans* pharynx and that 17,18-EEQ is a regulator of the *C. elegans* pharyngeal pumping activity. More recently, it was reported that *C. elegans* P450 13A12 and P450 reductase co-expressed in insect cells catalyze the epoxygenation of EPA into 17,18-EEQ and of AA into 14,15-epoxy-eicosatrienoic acid [58] (Table 3). The same article showed that 17,18-EEQ increases the *C. elegans* locomotion activity.

**Figure 2:**
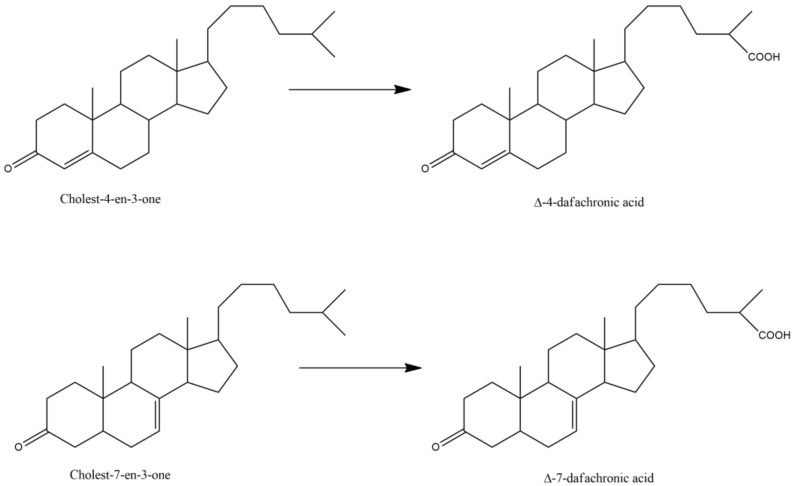
P450 22A1 (DAF-9) catalyzed oxidation of cholesten-3-ones into dafachronic acids.

Finally, the most documented *C. elegans* P450 is called DAF-9 or P450 22A1, with nearly 45 articles that refer to this protein. Indeed, P450 22A1 is involved in several pathways controlling dauer formation [29], life span, and gonadal migration [31,37]. P450 22A1 catalyzes a key step in the biosynthesis of 3-keto-cholestenoic acids, also called dafachronic acids, DAs (Table 3). Those bile acid-like steroids act as ligands of the DAF-12 nuclear receptor that governs *C. elegans* larval development and adult longevity [37,50,67]. DAF-9 catalyzes the oxidation of a terminal methyl group of the lateral chain of cholest-4-en-3-one or cholest-7-en-3-one with the formation of delta-4- and delta-7-dafachronic acids, respectively (Figure 2) [57,60]. DAF-9 is the equivalent of P450 27A1, the human P450 that catalyzes this oxidation of a terminal methyl group of the lateral chain of 3-keto-steroids in man.

Several redox systems are involved in the transfer of electrons from NADPH or NADH for dioxygen reduction at the P450 active site [68]. In all the above-mentioned oxidations catalyzed by microsomal type P450s, electrons from NADPH should be transferred to the P450 active site by a P450 reductase, analogous to human P450 reductase, that is encoded by the *C. elegans* emb-8 gene [69]. During embryonic development, emb-8 activity is essential for normal interactions between the pronucleus/centrosome complex and the posterior cortex and, thus, for proper anterior-posterior polarity. Emb-8 is also required for the formation of the secreted eggshell [69]. EMB-8 plays an important role in fatty acid modification. For instance, as mentioned in the previous paragraph, oxidation of EPA to 17,18-EEQ by adult worm microsomes is NADPH and P450 reductase dependent [47], and *C. elegans* P450 13A12 and P450 reductase co-expressed in insect cells catalyze the oxidation of EPA into 17,18-EEQ and of AA into 14,15-epoxy-eicosatrienoic acid [58].

The nature of the protein(s) responsible for electron transfer to the only *C. elegans* mitochondrial P450, P450 44A1, is less clear. In *C. elegans* mitochondrion, the Y62E10A.6 and Y73FBA.27 genes are coding for an adrenodoxin reductase and a ferredoxin, respectively [9]. If P450 44A1 functions as a genuine mitochondrial P450, those proteins would supply electrons to P450 44A1, even though it was argued that *C. elegans* lacks classical mitochondrial-type P450 [22]. More data on the nature and roles of P450 44A1 are required to answer those questions.

## 4. P450s of *C. elegans* and Xenobiotics

### 4.1. Induction of C. elegans P450s by Xenobiotics

As shown in Table 4, many *C. elegans* P450s are induced after the exposition of the worm to various xenobiotics. Thus, ethanol acts as an inducer of P450s 13A12, 13B1, 25A1, 25A2, 29A2, 32B1, 33B1, 33C6, 34A4, 4A6, 35A3, 35A5, 35B1, 35B2, 35C1, 36A1 and 37B1 [70,71], and caffeine is an inducer of P450s 13A8, 13A12, 14A1, 14A2, 14A4, 14A5, 32A1, 33C3, 33C4, 33C6, 33C7, 33C9, 33E1, 33E2, 33E3, 33E4, 34A7, 34A9, 35A2, 35A3, 35A4, 35A5, 35B1, 35B2, and 43A1 [72]. Interestingly, drugs such as primaquine and lansoprazole are inducers of several members of family 35 (35A1, A2, A3, A4, A5, B1, B2, and C1) and P450 31A3 [73,74,75], whereas rifampicin is an inducer of P450 13A7 [76]. Many pesticides, such as atrazine, fenitrothion, DDT, dichlorvos, rotenone, and thiabendazole are inducers of members of family 35 [73,74,75,77,78,79], whereas endosulfan, cypermethrin, chlorpyrifos, rotenone, and dichlorvos are inducers of P450 34A9 [77], and glyphosate and paraquat of P450 29A2 [77]. Aromatic and polyaromatic molecules, such as beta-naphthoflavone, fluoranthene, bisphenol A, and polychlorinated biphenyls (PCBs), act as inducers of several members of family 35 [56,73,74,75,80,81,82]. PCBs are anthropogenic molecules that were produced to be used as dielectric and coolant fluids in electrical devices. They are highly stable and therefore can accumulate in fat tissues. PCB 52 is one among many congeners. It is noteworthy that PCB 52 is also an inducer of P450s 34A6, 14A3, and 14A5 [56,75,83]. Ethidium bromide is an inducer of several members of families 33 and 35 [84,85]. Metal ions related to Cd are inducers of P450s 13A4,13A5, 13A6, 13A7, 14A4, 29A2, 33C5, 33C7, 34A9, and 35A2 [86], whereas those related to Zn, Cu, and Al induce P450s 29A2, 34A9, and 35A2 [87]. Other organic compounds have also been described as inducers of several P450s of *C. elegans* (Table 4) [88,89,90,91,92].

It is also noteworthy that an endogenous molecule such as progesterone acts as an inducer of P450s 25A2, 25A6, 29A2, 37A1, and 37B1 [93].

### 4.2. Metabolism of Xenobiotics by C. elegans P450s

Several Phase I and Phase II enzymes involved in xenobiotics metabolism in most living organisms, such as P450-dependent monooxygenases, dehydrogenases, UDP-glucuronyl transferases, and glutathione transferases, are present in *C. elegans* [95]. They are upregulated in long-lived *C. elegans* dauer larva [35]. As they are involved in the metabolism and elimination of potentially toxic endobiotics and xenobiotics, these data suggested that those compounds may be the major determinants of molecular damages that cause aging in *C. elegans* [35].

**Figure 3:**
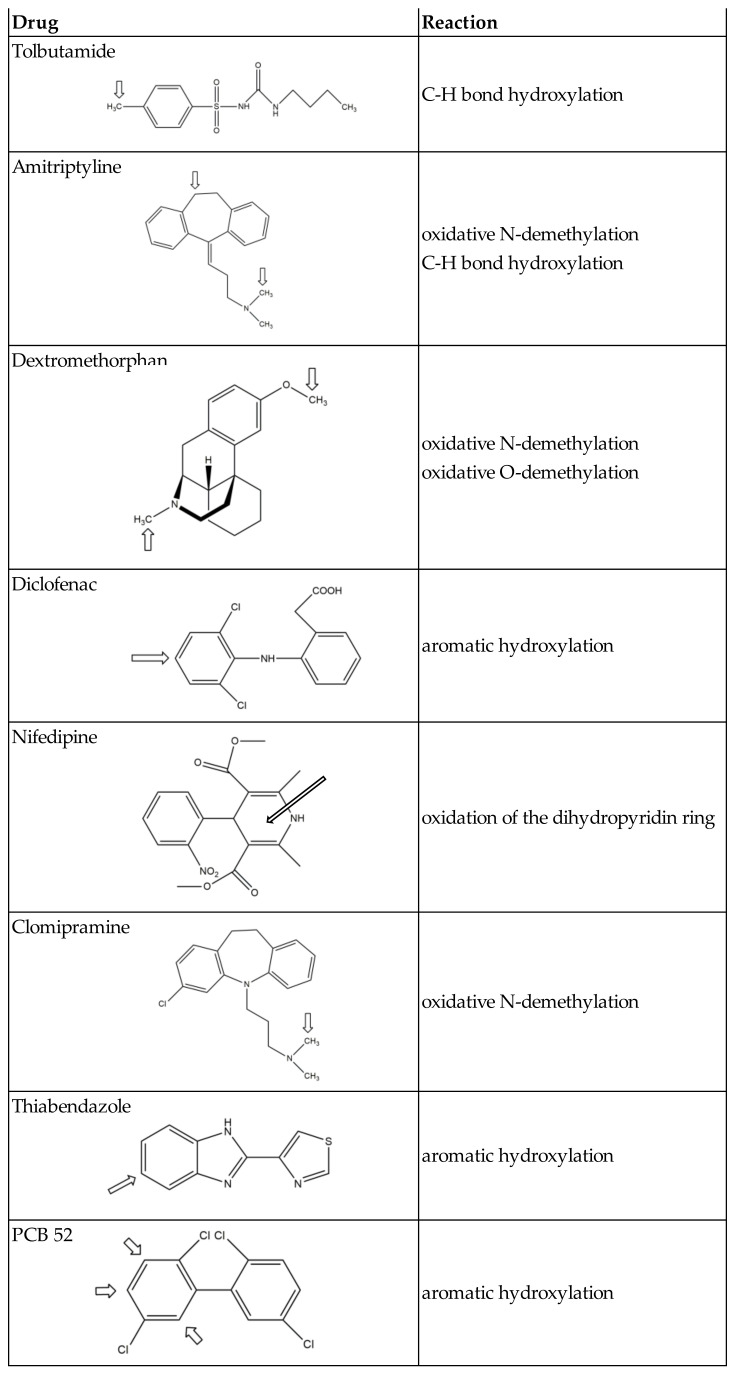
Oxidations of xenobiotics by *C. elegans* (arrows indicate the oxidation site(s)).

First results showing that *C. elegans* P450s are responsible for the metabolism of xenobiotics were concerned with a worldwide distributed pollutant, 2,2′,5,5′-tetrachloro-biphenyl, PCB 52 [83]. Metabolism of PCB 52 by *C. elegans* leads to C3- and C4- and/or C6-hydroxy-PCB 52. Experiments using RNAi and P450- mutant strains showed that P450 34A6 and members of the P450 14A family were involved in these aromatic hydroxylations (Table 5 and Figure 3). A few years later, it was reported that *C. elegans* hydroxylates thiabendazole in position 5 of its aromatic ring and that P450 35D1 is responsible for this reaction [96] (Table 5 and Figure 3).

Even more recently, a study of the metabolism by *C. elegans* of a series of drugs well known to be oxidized in humans by P450s of families 1, 2, and 3 was published [97]. Tolbutamide, amitriptyline, dextromethorphan, diclofenac, nifedipine, and clomipramine that are oxidized by P450s of families 2 and 3 in humans were found to undergo similar oxidations by *C. elegans*. However, phenacetin that is oxidized into paracetamol by P450 1A2 in humans is not oxidized by *C. elegans*. These data would indicate the lack of equivalents of P450s of family 1 in *C. elegans* [97]. Experiments of P450 gene inactivation by RNAis showed that tolbutamide hydroxylation by *C. elegans* is mainly dependent on P450 34A9 and to a lesser extent on P450s 36A1 and 34A10 (Table 5). Those P450s are the homologs of P450s of family 2 (2C8, 2C9, and 2C19) that are responsible for this hydroxylation in humans. However, the oxidative N-demethylation and C-H bond hydroxylation of amitriptyline were found to be catalyzed by several *C. elegans* P450s are the equivalents of human P450s of families 2,3, and 4, whereas those reactions are mainly catalyzed by P450 2D6 in humans [97].

Other experiments have shown that some P450s play a role in the development of toxic effects of several xenobiotics for *C. elegans*. Thus, the DNA damages and growth inhibition caused by aflatoxin B1, a mycotoxin, in *C. elegans* were reported to depend on the P450 reductase of this organism, even though the involved P450s were not determined [98] (Table 6). The toxic effects of fenitrothion for *C. elegans* also appear to derive from the formation of reactive metabolites mainly by P450 35A2 [79] (Table 6). Quite recent data have shown that the genotoxic effects of benzo(a)pyrene, B(a)P on *C. elegans* do not depend on P450s equivalent to those of human family 1A that do not seem to exist in *C. elegans* but depend on P450s 35A2, 35A3, and 35A5 [99].

On the contrary, *C. elegans* P450s may be involved in the detoxication of some xenobiotics. This is the case of P450 13A7, an equivalent of human P450 3A4, that mitigates tetrabromobisphenol A-induced toxicity for *C. elegans* [94]. This is also the case of P450s of the 35 family, P450s 35A1, 35A2, 35A4, 35B3, and 35C1, that are responsible for the detoxication of 3-bromopyruvate [100] (Table 6). Finally, it was recently reported that the expression of Zebrafish P450 1A1 in *C. elegans* protects it from the toxic effects of B(a)P and other polyaromatic hydrocarbons [101].

## 5. Conclusions

The *C. elegans* genome contains 76 genes coding for P450s. Phylogenetic analysis shows that they are homologous to mammalian P450s of families 2, 3, and 4. Many compounds, including drugs, pesticides, polyaromatic molecules, metal ions, ethanol, or caffeine, act as inducers of *C. elegans* P450s (Table 2). Very few data about the biochemical properties of those P450s have been published so far. Thus, no spectroscopic or structural study of those P450s has been reported so far. However, an increasing number of articles have appeared on reactions catalyzed by them in the metabolism of xenobiotics (Table 5 and Table 6 and Figure 3) and of endobiotics such as fatty acid derivatives and steroids (Table 3 and Figure 2). Moreover, the implication of some P450s in various biological functions of *C. elegans* without mention of the precise reaction catalyzed by those P450s has been reported in several articles (Table 2). These preliminary studies at the biological level may pave the way to decipher the enzymatic reactions catalyzed by these P450s. Invalidation of the expression of several P450s has shown their implication in *C. elegans* survival, morphology, embryonic development, growth, larval development, dauer formation, life span, reproduction, movement, pharyngeal pumping, fat content, or lipid composition and metabolism. All the data reported so far for each P450 are schematically summarized in Table 7. Moreover, because of the importance of the use of *C. elegans* as a model in biology and toxicology, more complete knowledge of its P450s appears to be important in the near future.

## Figures and Tables

**Table 1:** Genes and pseudogenes (in italics) of P450s in *C. elegans*.

Family	Transcript	Transcript Length (nt)	Protein	Protein Length (aa)
13	T10B9.8.1	1771	CYP-13A1	519
	T10B9.7.1	2258	CYP-13A2	515
	T10B9.5.1	1677	CYP-13A3	520
	T10B9.1.1	1655	CYP-13A4	520
	T10B9.2.1	1665	CYP-13A5	520
	T10B9.3.1	1759	CYP-13A6	518
	T10B9.10.1	1623	CYP-13A7	518
	T10B9.4.1	1594	CYP-13A8	509
	*T10B9.6*	*1423*	*CYP-13A9*	
	ZK1320.4.1	1680	CYP-13A10	519
	F14F7.2.1	1653	CYP-13A11	517
	F14F7.3.1	1726	CYP-13A12	518
	F02C12.5.1	1652	CYP-13B1	510
	K06G5.2.1	1786	CYP-13B2	511
14	K09A11.2.1	1533	CYP-14A1	491
	K09A11.3.1	1535	CYP-14A2	492
	K09A11.4.1	1541	CYP-14A3	498
	R04D3.1.1	1549	CYP-14A4	491
	F08F3.7.1	1561	CYP-14A5	492
22	T13C5.1	1999	CYP-22A1 (daf-9)	572
23	B0304.3.1	1719	CYP-23A1	534
25	C36A4.1	1631	CYP-25A1	502
	C36A4.2	1570	CYP-25A2	502
	C36A4.3	1775	CYP-25A3	502
	C36A4.6	1677	CYP-25A4	501
	*F42A6.4*	*1506*	*CYP-25A5*	
	K06B9.1	708	CYP-25A6	236
29	*C44C10.2*	*1515*	*CYP-29A1*	
	T19B10.1	1733	CYP-29A2	503
	Y38C9B.1	1743	CYP-29A3	503
	B0331.1	1682	CYP-29A4	502
31	*C01F6.3*	*1389*	*CYP-31A1*	
	H02I12.8	1631	CYP-31A2	495
	Y17G9B.3	1597	CYP-31A3	495
	Y62E10A.15b2 3	1077	CYP-31A5	308
32	C26F1.2	1691	CYP-32A1	529
	Y5H2B.5	1648	CYP-32B1	516
33	C12D5.7	1591	CYP-33A1	492
	C25E10.21	1491	CYP-33B1	496
	C45H4.21	1739	CYP-33C1	495
	C45H4.17a2	1585	CYP-33C2	495
	F41B5.4	1784	CYP-33C3	500
	F44C8.1	1583	CYP-33C4	493
	F41B5.3a.1	1568	CYP-33C5	494
	F41B5.7a1	1778	CYP-33C6	494
	F41B5.4	1560	CYP-33C7	494
	R08F11.3	1667	CYP-33C8	494
	C50H11.15	1616	CYP-33C9	496
	Y49C4A.9	1738	CYP-33C11	495
	Y5H2B.61	2035	CYP-33C12	426
	K05D4.4	1597	CYP-33D1	492
	Y17D7A.41 2	1579	CYP-33D3	495
	C49C8.41	1753	CYP-33E1	494
	F42A9.51	1687	CYP-33E2	494
	F42A9.4	890	CYP-33E3	236
34	T10H4.10	1595	CYP-34A1	504
	T10H4.11	1766	CYP-34A2	502
	*C41G6.1*	*1481*	*CYP-34A3*	
	T09H2.1.1	1680	CYP-34A4	500
	B0213.10	1591	CYP-34A5	499
	B0213.11	1722	CYP-34A6	498
	B0213.12	1579	CYP-34A7	499
	B0213.14	1584	CYP-34A8	499
	B0213.15	1665	CYP-34A9	516
	B0213.16	1571	CYP-34A10	499
35	C03G6.14	1546	CYP-35A1	494
	C03G6.15	1588	CYP-35A2	494
	K09D9.2	587	CYP-35A3	494
	C49G7.8	1552	CYP-35A4	494
	K07C6.5	1727	CYP-35A5	494
	K07C6.4	1648	CYP-35B1	499
	K07C6.3	1621	CYP-35B2	499
	K07C6.2	1500	CYP-35B3	499
	C06B3.3	1534	CYP-35C1	495
	F14H3.10	1576	CYP-35D1	499
	*F14H3.13*	*558*	*CYP-35D2*	
36	C34B7.3	1750	CYP-36A1	493
37	F01D5.9	1561	CYP-37A1	508
	F28G4.1	1736	CYP-37B1	509
42	Y80D3A.5	1826	CYP-42A1	511
43	E03E2.1	1698	CYP-43A1	526
44	ZK177.5	1551	CYP-44A1	489

**Table 2:** Implication of P450s in biological functions of *C. elegans*.

Biological Functions	P450s	Ref.
Survival/life span	22A1	[29,30,31,32,33,34,35,36]
Morphology	22A1, 33E2	[37,38,39,40,41]
Embryonic development	22A1,31A2, 31A3	[37,42,43]
Larval development	22A1, 32A1, 32B1, 33B1, 33C1, 33C2, 33D1	[35,37,42,44]
Dauer formation	13A5, 13A7, 14A1, 14A3, 22A1, 29A3, 32A1, 32B1, 33B1, 33C1, 33C2, 33D1, 33E2, 34A2, 34A5, 34A6, 34A7, 34A8, 34A9, 35B1, 35B2	[29,30,31,35,45,46,47,48,49,50]
Reproduction	31A2, 31A3, 35A2	[31,42,51,52,53]
Fat content	29A3, 31A2, 31A3, 33E2, 35A1, 35A2, 35A3, 35A4, 35A5	[42,47,48,51,53,54,55,56]
Lipid metabolism	13A12, 22A1, 29A3, 31A2, 31A3, 33E2, 35A1, 35A2, 35A4, 35A5, 37B1	[32,40,42,47,48,50,51,54,55,57,58,59,60,61]

**Table 3:** Implication of P450s in the metabolism of endogenous compounds in *C. elegans*.

Endobiotics	Metabolite(s)	Reaction(s)	P450(s) Involved	Ref.
EPA	17,18-epoxy-eicosatetraenoic acid	Epoxidation	13A12, 29A3, 33E2	[47,55,58]
AA	19-hydroxy-AA	C-H bond hydroxylation	33E2	[55]
AA	14,15-epoxy-eicosatrienoic acid	Epoxidation	13A12	[58]
Cholesten-3-ones	Dafachronic acids	Oxidation of CH_3_ to COOH	22A1 (DAF-9)	[57,60]

**Table 4:** Induction of *C. elegans* P450s by xenobiotics.

Chemical Class	Compounds	P450s Induced	Ref.
Alcohol	Ethanol	13A12, 13B1, 25A1, 25A2, 29A2, 32B1, 33B1, 33C6, 34A4, 34A6, 35A3, 35A5, 35B1, 35B2, 35C1, 36A1, 37B1	[70,71]
Alkaloid	Caffeine	13A8, 13A12, 14A1, 14A2, 14A4, 14A5, 32A1, 33C3, 33C4, 33C6, 33C7, 33C9, 33E1, 33E2, 33E3,33E4, 34A7, 34A9, 35A2, 35A3, 35A4, 35A5, 35B1, 35B2, 43A1	[72]
Aromatic compound	Ethidium bromide, 2,2′,5,5′-tetrachlorobiphenyl (PCB52)	13A7, 14A3, 14A5, 33C3, 33C4, 33C5, 33C6, 33C7, 33D3, 34A6, 35A1, 35A2, 35A3, 35A4, 35A5, 35B1, 35B2, 35B3, 35C1	[56,73,74,83,84,85]
Drug	Rifampicine, Lansoprazole, primaquine, phenobarbital	13A7, 31A3, 35A1, 35A2, 35A3, 35A4, 35A5, 35C1	[73,74,75,76]
Hormone	Progesterone, 17-β-estradiol	25A2, 25A6, 29A2, 37A1, 37B1	[93]
Metal ion	Zinc, mercury, copper, arsenic, aluminum, cadmium	13A4, 13A5, 13A6, 13A7, 14A4, 29A2, 33C5, 33C7, 34A9, 35A2	[86,87]
Nanoparticle	Silver and titane nanoparticles	35A2	[53,89]
Organic compound	Acrylamide, pyrazole	13A12; 31A1, 31A3	[73,90]
Pesticide	Glyphosate, paraquat, endosulfan, cyperméthrine, chlorpyrifos, dichlorvos, dichlorodiphenyltrichloroethane (DDT), pyridazine, rotenone, atrazine, fenitrothion, thiabendazole	22A1, 29A2, 34A9, 35A1, 35A2, 35A5, 35B1, 35B2, 35B3, 35C1, 35D1	[73,74,75,77,78,79]
Phtalate	Diethylhexylphtalate (DEHP)	35A2	[91]
Polycyclic aromatic hydrocarbons	Beta-naphthoflavone, fluoranthene	14A5, 35A1, 35A2, 35A3, 35A4, 35A5, 35B1, 35B2, 35C1, 37B1	[73,74,75]
Phenol/Polyphenol	Bisphenol A, tetrabromobisphenol A, resveratrol	13A6, 13A7, 35A2	[80,81,82,92,94]

**Table 5:** Metabolism of xenobiotics by *C. elegans*: implication of P450s.

Xenobiotic	Metabolite(s)	Reaction(s)	P450(s) Involved	Ref.
2,2′,5,5′-tetrachlorobiphenyl (PCB52)	3-,4- or 6- hydroxy-PCB52	Aromatic hydroxylations	14A members, 34A6	[83]
Thiabendazole (TB)	5-hydroxy-TB	Aromatic hydroxylation	35D1	[96]
Tolbutamide (TA)	Hydroxy-TA	C-H bond hydroxylation	34A9, 34A10 and 36A1	[97]
Amitriptyline	Nortriptyline E-10-hydroxyamitriptyline	Oxidative *N*-demethylation C-H bond hydroxylation	Many P450s n.d.	[97]
Dextromethorphan	Dextrorphan 3-methoxymorphinan	*O*-demethylation *N*-demethylation	Many P450s n.d.	[97]
Diclofenac	4′-hydroxy-diclofenac	Aromatic hydroxylation	n.d.	[97]
Nifedipine	Oxidized nifedipine	Dehydrogenation	n.d.	[97]
Clomipramine	Norclomipramine	*N*-demethylation	n.d.	[97]

n.d. not determined.

**Table 6:** Implication of P450s in the biological effects of xenobiotics on *C. elegans*.

Xenobiotic	Biological Effect(s)	P450(s) Involved	P450 Role	Ref.
Aflatoxine B1	DNA damage, growth inhibition	n.d. P450 reductase involved	Toxic activation	[98]
Tetrabromobisphenol A	Toxicity	13A7	Detoxication	[94]
3-bromopyruvate	Toxicity	35A1, 35A2, 35A4, 35B3, 35C1	Detoxication	[100]
Fenitrothion	Toxicity	35A2	Toxic activation	[79]
Benzo(a)pyrene	Toxicity	35A2, 35A3, 35A5	Toxic activation	[99]

n.d. not determined.

**Table 7:** Data presently reported on each *C. elegans* P450.

Family	P450s	Endogenous Function(s)	Inducers	Xenobiotic(s) Metabolized	Ref.
13	13A1	-	-	-	-
	13A2	-	-	-	-
	13A3	-	-	-	-
	13A4	-	Cadmium	-	[86,102]
	13A5	Dauer larvae and long-lived state	Cadmium	-	[35,86]
	13A6	-	Cadmium Resveratrol	-	[86,92]
	13A7	Dauer larvae and long-lived state	Ethidium Bromide Cadmium Rifampicine	Tetrabromobisphenol A	[35,76,84,86,94]
	13A8	-	Caffeine	-	[72]
	13A10	-	-	-	-
	13A11	-	-	-	-
	13A12	Lipid metabolism/fat content	Acrylamide Caffeine Ethanol	-	[58,71,72,90]
	13B1	-	Ethanol	-	[71]
	13B2	-	-	-	-
14	14A1	Dauer larvae and long-lived state	Caffeine	PCB52	[35,72,83]
	14A2	-	Caffeine	PCB52	[72,83]
	14A3	Dauer larvae and long-lived state	PCB52	PCB52	[35,56,83]
	14A4	-	Caffeine Cadmium	PCB52	[72,83,86]
	14A5	-	Beta-naphthoflavone Caffeine PCB52	PCB52	[72,75,83]
22	22A1 (DAF-9)	Survival/life span morphology Embryonic development Larval development/dauer formation Lipid metabolism/fat content	Atrazine	-	[29,30,31,32,33,34,35,36,37,38,39,40,41,42,43,44,45,46,47,48,49,50,55,57,60,62,75]
23	23A1	-	-	-	-
25	25A1	-	Ethanol	-	[71]
	25A2	-	Ethanol Progesterone	-	[70,71,93]
	25A3	-	-	-	-
	25A4	-	-	-	-
	25A6	-	Progesterone	-	[93]
29	29A1	-	-	-	-
	29A2	-	Ethanol Progesterone Aluminum, cadmium, copper, zinc Glyphosate, paraquat, dichlorvos, rotenone	-	[71,77,86,93]
	29A3	Dauer larvae Lipid metabolism/fat content	-	-	[47]
	29A4	-	-	-	-
31	31A2	Embryonic development Reproduction Lipid metabolism/fat content	-	-	[35,48,97,103]
	31A3	Embryonic development Lipid metabolism/fat content	Atrazine Lansoprazole, phenobarbital, primaquine Pyrazole, toluene	-	[73,97,103]
	31A5	-	-	-	
32	32A1	Larval development	Caffeine	-	[35,72]
	32B1	Larval development/dauer formation	Ethanol	-	[35,70]
33	33A1	-	-	-	-
	33B1	-	-	-	-
	33C1	Larval development/dauer formation	-	-	[35]
	33C2	Larval development/dauer formation	-	-	[35]
	33C3	-	Caffeine Ethidium bromide	-	[72,84]
	33C4	-	Caffeine Ethidium bromide	-	[72,84]
	33C5	-	Cadmium Ethidium bromide	-	[84,86]
	33C6	-	Caffeine Ethanol Ethidium bromide	-	[70,72,84]
	33C7	-	CadmiumCaffeine Ethidium bromide	-	[70,72,84]
	33C8	-	-	-	-
	33C9	-	Caffeine	-	[72]
	33C11	-	-	-	-
	33C12	-	-	-	-
	33D1	Larval development/dauer formation	-	-	[35]
	33D3	-	Ethidium bromide	-	[84]
	33E1	-	Caffeine	-	[72]
	33E2	-	Caffeine	-	[72]
	33E3	Morphology Dauer formation Lipid metabolism/fat content	Caffeine	-	[35,47,48,55,72]
	33E4	-	Caffeine	-	[72]
34	34A1	-	-	-	-
	34A2	Dauer formation	-	-	[35]
	34A4	-	Ethanol	-	[70]
	34A5	Dauer formation	-	-	[35]
	34A6	Dauer formation	Ethanol	PCB52	[35,70,83]
	34A7	Dauer formation	Caffeine	-	[35,72]
	34A8	Dauer formation	-	-	[35]
	34A9	Dauer formation	Caffeine Arsenic, cadmium, nickel, zinc	Tolbutamide	[35,72,77,86,97]
	34A10	-	-	Tolbutamide	[97]
35	35A1	Lipid metabolism/fat content	Atrazine Beta-naphthoflavone, fluoranthene Caffeine Ethidium bromide, PCB52 Lansoprazole, primaquine	3-bromopyruvate	[54,56,73,74,75,84,100]
	35A2	Reproduction Lipid metabolism/fat content	Aluminum, arsenic, cadmium, copper, mercury, zincAtrazine, dichlorvos, dichlorodiphenyltrichloroethane (DDT), rotenone, fenitrothion Beta-naphthoflavone, fluoranthene Bisphenol A CaffeineEthidium bromide, PCB52 N-Nitrosodiethylamine (NDMA), dibromoacetic acid (DBAA), Lansoprazole, primaquine Silver and titane nanoparticles	3-bromopyruvate, fenitrothion, B(a)P	[32,53,54,56,72,73,74,77,79,80,81,82,86,87,89,91,100]
	35A3	Lipid metabolism/fat content	Beta-naphthoflavone Caffeine Ethanol PCB52 Lansoprazole, primaquine	B(a)P	[56,71,72,73,84]
	35A4	Lipid metabolism/fat content	Beta-naphthoflavone Caffeine Lansoprazole, primaquine PCB52	3-bromopyruvate	[56,72,73,100]
	35A5	Lipid metabolism/fat content	Atrazine Beta-naphthoflavone, fluoranthene Caffeine Ethanol Ethidium bromide, PCB52 Lansoprazole, primaquine	B(a)P	[56,71,72,73,74,75,84]
	35B1	Dauer formation	Atrazine Beta-naphthoflavone Caffeine Ethanol Ethidium bromide Lansoprazole	-	[35,71,72,73,75,84]
	35B2	Dauer formation	Atrazine Beta-naphthoflavone Caffeine Ethanol Ethidium bromide Lansoprazole, primaquine	-	[35,71,72,73,75,84,85]
	35B3	-	Ethidium bromide	3-bromopyruvate	[84,100]
	35C1	-	Atrazine Beta-naphthoflavone, fluoranthene EthanolLansoprazole, primaquine PCB52	3-bromopyruvate	[71,73,74,75,100]
	35D1	-	Thiabendazole	Thiabendazole	[78,96]
36	36A1	-	Ethanol	Tolbutamide	[71,97]
37	37A1	Lipid metabolism/fat content	Progesterone	-	[93,104]
	37B1	Lipid metabolism/fat content	Ethanol Fluoranthene Progesterone, 17-ß-estradiol	-	[59,70,75,93]
42	42A1	-	-	-	-
43	43A1	-	Caffeine	-	[72]
44	44A1	-	-	-	-

## Data Availability

Not applicable.

## Abbreviations

17:18-EEQ: 17,18-epoxy-eicosatetraenoic acid; AA, arachidonic acid; AhR, aryl hydrocarbon receptor; ARNT, aryl hydrocarbon receptor nuclear translocator; BaP, benzo(a)pyrene; *C. elegans*, *Caenorhabditis elegans*; DAs, dafachronic acids; DDT, dichlorodiphenyltrichloroethane; EPA, eicosapentaenoic acid; KO, knock-out; LD50, lethal dose 50; P450, cytochrome P450; PCB 52, 2,2′,5,5′-tetrachloro-biphenyl.
